# Antiseptics and Disinfectants Used in Dental Surgery

**Published:** 1892-02

**Authors:** Savill H. Hayward


ARTICLE II.

### ANTISEPTICS AND DISINFECTANTS USED IN DENTAL SURGERY.

BY SAVILL H. HAYWARD.

A Paper read before the Students' Society, Dental Hospital of London.

*Mr. President aud Gentlemen :*

I cannot pretend that in the paper I have the honor of reading to-night I shall be able to put before you anything of an original nature, but if I can give a brief description of the physical characteristics, composition, properties, and uses of some of the antiseptics and disinfectants used in dental surgery, with a few suggestions as to the best means of exhibiting them, it may perhaps serve a useful purpose. I was encouraged to write this paper by the assurance that the paper itself was of secondary importance, provided it gave rise to a good discussion, an end I think guaranteed almost by the nature of its subject, since it is one which can hardly fail at all times to be of the greatest possible interest to the dental surgeon and the dental student, the more so since each year greater light is being thrown on the subject of micro-organisms, and our scientific men are confirming and elucidating the germ theory of disease, pressing home upon the surgeon the conviction that success in his operations very largely depends upon the clearness with which he realizes the necessity of his surgery being of a thoroughly antiseptic nature.

It is not possible to draw a line of demarcation between the two classes of antizymotics, viz., antiseptics and disinfectants, the same substance frequently possessing properties entitling it to a place in both. I shall not, therefore, speak of them as separate classes, and though the following may be far from a perfect, yet it may be I think, a convenient method of arrangement in which to discuss them. (I.) Those which owe, and (II.) those which do not owe their antiseptic and disinfecting powers to certain contained gases, in solution or combination, which they evolve under ordinary or special conditions; and (III.) it will perhaps be convenient to describe separately the essential oils.

In the first division we shall find peroxide of hydrogen, ozonic ether, permanganate of potash, sulphurous acid, and the preparations of chlorine e.g.: liq. sodii chlorinata, and liq. chlori.

*Peroxide of Hydrogen* or Hydroxyl in aqueous solution is a clear, inodorous, watery liquid, prepared by adding hydrated peroxide of barium to dilute sulphuric acid, removing the precipitate, neutralizing with baryta water and again filtering; the solution of hydroxyl thus obtained is nearly pure. For medical purposes the solution is made of 10 volume strength, *i. e.*, when decomposed 1 c. c. will yield 10 c. c. of free oxygen, but it can also be obtained of 12 and 20 volume strength. It has a harsh, somewhat bitter taste, and is possessed of very considerable oxidising powers; and has a well-marked action on bacteria, a solution of 1:8000 preventing their development (Miller). The second atom of oxygen in its composition is very loosely combined, and is quickly liberated in the active state when brought into contact with certain organic matters, or any animal substance undergoing putrefaction; on this account it is very valuable in the treatment of pulpless teeth for the syringing out of the pulp chambers and root canals, and as an injection for alveolar abscess, the peroxide continuing to froth briskly by the escape of gas as long as any unoxidised putrefactive material is left. Two cases are recorded in the *Lancet*, of 1889, of necrosis of the jaws, in which, after removal of a sequestum the discharge of pus from the retained parts was stopped, and the parts healed rapidly under the use of injections of peroxide, and the same surgeon has recorded his successful treatment by the same method of a case of pyorrhœa alveolaris. The same line of treatment in pyorrhœa has also been followed in America with very good results by Drs. Aingle and Martindale. It has the drawback of being a very unstable compound, and should be used as fresh as possible and preserved in closely stoppered blue bottles protected from light and heat, by both of which its decomposition is effected.

Equally efficacious with the above and with the advantage of being very stable is ozonic ether, *i.e.*, ether containing $H_2$ $O_2$, in 30 vol. strength, with some alcohol. It is miscible with water, and in addition to its antiseptic properties is said to have a stimulating effect upon ulcerated surfaces. Since by the liberation of one atom of oxygen, peroxide of hydrogen is reduced to water, it is evidently quite useless as a permanent or temporary dressing, and as a sterilizer of carious dentine, Miller has proved its uselessness from the same causes, except when the amount of dentine to be acted on is very minute. As a devitalizer of the bacteria of the mouth he found the 10 per cent. solution to be equivalent to a solution of carbolic acid 1 : 100, sterilizing the liquid containing the micro-organisms in from ten to fifteen minutes.

*Permanganate of Potash*, obtained by the action of boiling water upon manganate of potash occurs as slender acicular crystals of a deep purple color, freely soluble in water. Its solution has no disagreeable odor, and by the ease with which it parts with oxygen the permanganic radicle being reduced to the black oxide, it is enabled to destroy the organisms producing sepsis or fermentation, or forms chemical compounds incapable of decomposition with the materials on which they flourish, and by oxidizing the products of decomposition already formed so alters their chemical properties as to deodorize them. A solution of the strength of 1 : 1000 prevents the development of bacteria, though a much higher concentration is necessary to destroy the vitality of the spores. Its use is indicated in the treatment of pulpless teeth and abscesses, and as a mouth lotion in cases where the oral secretions are offensive, or there is purulent discharge in alveolar periostitis, salivation, etc.

The official solution, the liq. potassii permanganas, is of 1 per cent. strength, this diluted till it is about 1 : 1000 makes a solution suitable for syringing root canals, mouth lotions, etc.; for the *dressing* of root canals a much greater strength should be used, 1 : 200 or 250. Although less generally in use, the permanganate of calcium is preferable to the potash salt since it has not the extremely disagreeable taste of the latter.

Chlorine in the form of an aqueous solution, either of the gas itself or of one of the compounds in which it is in a loose state of combination, furnishes us with a most powerful disinfectant. Its action in this direction would appear to depend upon its great affinity for hydrogen and its consequent power of decomposing organic particles, nascent oxygen being set free and the chlorine further combining with the resulting broken down molecule. As a disinfectant it is greatly superior to carbolic acid, but has but little effect on bacteria. It is extremely useful in either of the forms mentioned as a mouth lotion in correcting the fœtor arising from an unhealthy condition of the mucous membrane, in mercurial salivation, cancrum oris, etc. The two official solutions used are the liq. chlori, a simple solution of rather less than 1 per cent. of the gas in water, and the liq. sodæ chlorinatæ, which is a mixture of hypochlorite of soda, chloride of sodium, and carbonate of soda in solution, and having 2·5 per cent. of available chlorine. The liq. sodæ chlorinatæ is a more stable preparation than the liq. chlori, and, since chlorine in the presence of water decomposes the latter with formation of hydrochloric acid; if liq. chlori is prescribed, it should be ordered to be freshly prepared. Both of these solutions when used should be largely diluted with water in the proportions of 1 : 12 or 16.—*Dental Record.*

*To be continued.*

